# THAT’S MY CREATOR’S REALM: UNDERSTANDING AFRICAN AMERICAN ASSISTED LIVING RESIDENTS’ END-OF-LIFE PREFERENCES

**DOI:** 10.1093/geroni/igz038.2371

**Published:** 2019-11-08

**Authors:** Candace L Kemp, Alexis A Bender, Tammie Quest, Mary H Coyle, Molly M Perkins

**Affiliations:** 1 Georgia State University, Atlanta, Georgia, United States; 2 Emory University, Atlanta, Georgia, United States; 3 Emory, Atlanta, Georgia, United States; 4 Emory University School of Medicine, Atlanta, Georgia, United States

## Abstract

Assisted living, one of the fastest growing formal long-term care options for older adults in the U.S., increasingly is a site for end-of-life care.. Most residents are non-Hispanic and white, yet African Americans reside in these settings and relatively little is known about their end-of-life preferences. In this paper, we present an analysis of data collected as part of a larger five-year mixed-methods NIA-funded study (R01AG047048) examining end of life in assisted living. We analyze longitudinal qualitative data collected over two years in a large (>90 beds) care community catering to African American older adults. Drawing on 850 hours of participant observation, in-depth interviews with 25 residents, and record review data, we seek to: (a) understand residents’ end-of-life preferences; and b) identify how and why preferences vary. Guided by principles of grounded theory, our analysis shows that most preferred a death where “you go to sleep and never wake up.” Yet, residents varied in their preferences for the timing and location of death, nature of end-of-life care, and use of advanced directives. Age, health, health literacy, perceived quality of life, and not wanting to be a burden all influenced preferences. For most, religious beliefs were a key factor shaping these preferences. Perceiving that end of life, including how, when, where one dies, and the nature of suffering and care, ultimately is their “creator’s realm,” led to the near universal conclusion: “I got no control over it.” We discuss implications of these findings for improving end-of-life care for African American residents.

